# New Species and Records of Scale Mites (Acariformes: Pterygosomatidae) from Geckos (Squamata: Gekkonidae and Caprodactylidae)

**DOI:** 10.1155/2018/9290308

**Published:** 2018-06-28

**Authors:** Monika Fajfer

**Affiliations:** Department of Animal Morphology, Faculty of Biology, Adam Mickiewicz University, Umultowska 89, 61–614 Poznan, Poland

## Abstract

Two new species of pterygosomatid mites parasitizing geckos of the families Gekkonidae and Caprodactylidae are described:* Geckobia africana* n. sp. from* Cnemaspis africana *(Werner) from Tanzania and* Geckobia milii* n. sp. from* Underwoodisaurus milii *(Bory De Saint-Vincent) from Australia. The former species is the most similar to* Geckobia loricata* Berlese, 1892, but differs by the presence of the propodonotal shield reduced to small platelets, slender and blunt-pointed setae in the posterolateral part of the idiosomal venter and the absence of thick serrate ventral setae in posterior part of the idiosoma. For the latter species and* Geckobia simplex* Hirst, 1926, a new species group is established based on the analysis of chaetotaxy of legs I–IV. Mites of this new species group differ from the* indica* group by the presence of five setae on tibiae I–III and setae* l'GI*,* l”GIV*,* dFI*,* dFII*, and* lFIII*. Species of the genus* Geckobia* are recorded from hosts of the genera* Cnemaspis *Strauch and* Underwoodisaurus *Wermuth for the first time.

## 1. Introduction

Mites of the family Pterygosomatidae Oudemans, 1910 (Acariformes: Prostigmata), are one of the most diverse group of permanent parasites of reptiles. They are represented by highly specific (mono- or stenoxeous) ectoparasites of lizards (Squamata: Sauria), with the exception of* Geckobia enigmatica* Bertrand and Pedrono, 2000, found on tortoises (Testudines: Testudinidae) [1], and species of the genus* Pimeliaphilus* Trägårdh, 1905, found on terrestrial arthropods [2]. To date, the family Pterygosomatidae includes 182 species grouped into nine genera recorded from all zoogeographical regions, except for the Antarctica [3, 4].

Within the Pterygosomatidae the genus* Geckobia* Mégnin, 1878, is the most species-rich with 73 species and subspecies. About two-thirds of the species are arranged into four species groups based on a trochanter-tibia chaetotaxy of legs I–IV [4, 5], i.e.,* latasti*,* haplodactyli*,* ovambica*, and* indica*, and into groups А, В based on differences in a tarsal chaetotaxy of legs I [4–6]. However, about one-third of the species of the genus* Geckobia *is not assigned to any of the groups due to their unique morphological characters or vague descriptions.

Currently, the genus is known from lizards of the families Gekkonidae, Phyllodactylidae, Carphodactylidae, Diplodactylidae, Eublepharidae, and Liolaemidae [3, 4]. Additionally, one species,* G. enigmatica*, have been found on the tortoise* Astrochelys yniphora* (Vaillant) [1]. The distribution of the genus is very wide, including six regions: the Palearctic, Afrotropic, Neotropic, Indomalayan, and Australasian ecozones [3, 4].

Below, I describe two new species of the genus* Geckobia* from the infraorder Gekkota:* Geckobia africana* n. sp. found on African gecko* Cnemaspis africana *(Werner) (Gekkonidae) from Tanzania and* Geckobia milii* n. sp. associated with thick-tailed gecko* Underwoodisaurus milii *(Bory De Saint-Vincent) (Carphodactylidae) from Australia. According to the leg chaetotaxy pattern, the former species belongs to* Geckobia* group 1 established by Jack [5] (the* latasti* group of Fajfer [4]) whereas for the latter species and* Geckobia simplex* Hirst, 1926, I propose a new species group based on its unique morphology. Species of the genus* Geckobia* are recorded herein from hosts of the genera* Cnemaspis *and* Underwoodisaurus *for the first time.

## 2. Materials and Methods

All mites were collected form geckos preserved in 70% ethanol in the Zoological Museum, University of Copenhagen, Denmark. The mites, before mounting in Hoyer's medium, were cleared and softened in Nesbitt's solution at 50°C for 1–3 hours. Then, the mites were studied using the light microscope Olympus BH-2 with differential interference contrast (DIC) illumination and drawings were made using a camera lucida drawing attachment. All measurements are given in micrometres as the data for the holotype followed by the ranges for the paratypes. Nomenclature of the leg and idiosomal setae follows Grandjean [7, 8], and names of the palpal setae follow Grandjean [9] as adapted to the family Pterygosomatidae by Bochkov & OConnor [10]. The scientific names of the lizards follow Uetz and Hošek [11].

Specimen depositories and reference numbers are cited using the following abbreviations: ZMUC, Zoological Museum, University of Copenhagen, Denmark; AMU, Adam Mickiewicz University, Department of Animal Morphology, Poznan, Poland. 

***Family Pterygosomatidae Oudemans, 1910***

***Genus Geckobia Mégnin, 1878***

***Species group latasti***

***Geckobia africana n. sp.***

*Type*-*Host*.* Cnemaspis africana *(Werner) (Sauria: Gekkonidae). 

*Type*-*Locality*. Tanzania, Iringa Region, Mufindi district, Uzungwa Scarp Forest Reserve, 8°31.58′S; 35°54′E; 8.III.1996, coll. McKamey. 

*Type Material*. Female holotype and 1 female paratype. Mites removed by M. Fajfer. Female holotype is deposited in the AMU (Reg. No. AMU-PTE26.1), female paratype in the ZMUC (Reg. No. R341385). 

*Etymology*. The species name is derived from the species name of the host and is a noun in apposition. 

**Figure 1:**
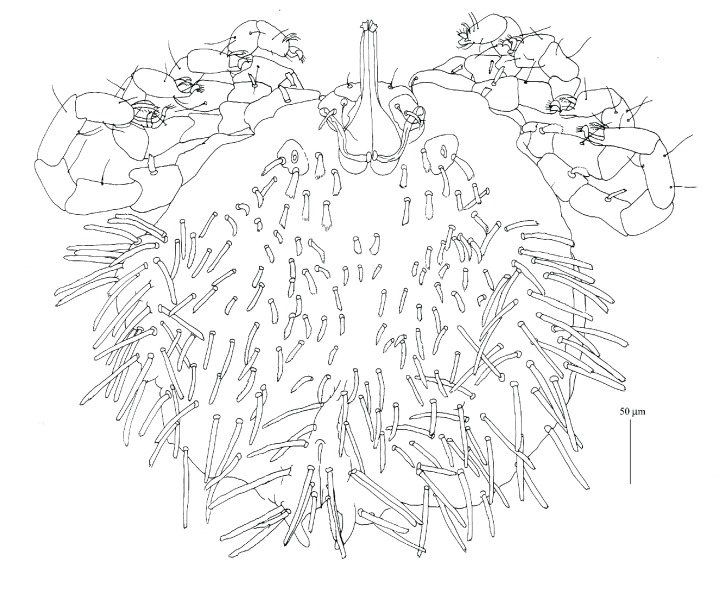
*Geckobia africana* n. sp., female in dorsal view.

**Figure 2:**
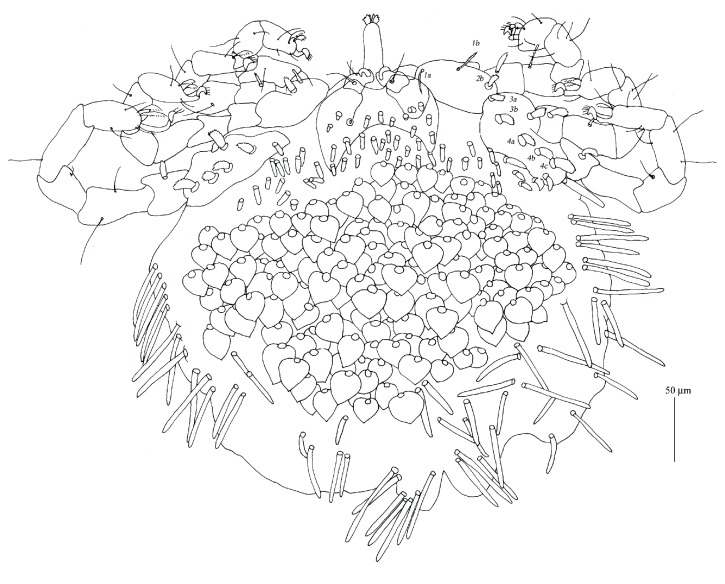
*Geckobia africana* n. sp., female in ventral view.

**Figure 3:**
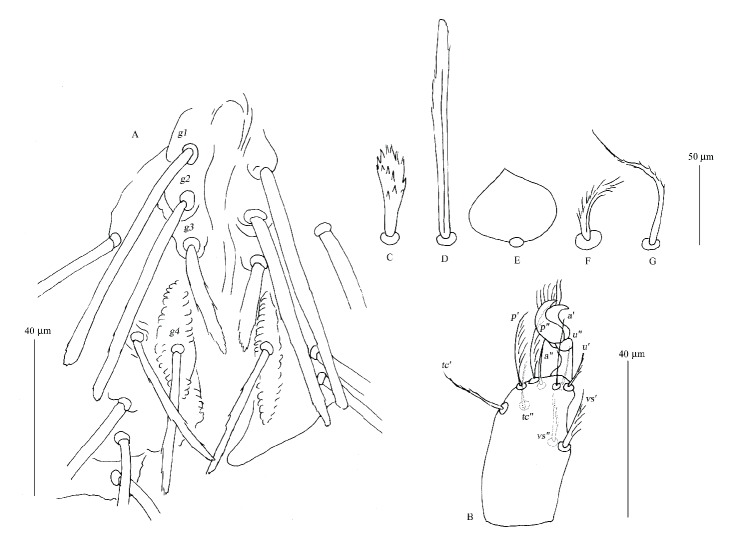
*Geckobia africana* n. sp., female, details. A, genital area, enlarged; B, tarsi III in lateral view; C, anterior dorsal seta; D, posterior dorsal seta; E, medial ventral seta; F, palp femoral seta* dF*; G, palp genual seta* dG*.

*Description (Figures 1–3)*

*Female*.* Gnathosoma*. Chelicerae 120 (125) long; basal swollen part 50 (55) long, slender distal part 70 (70) long. Movable cheliceral digit three-pronged. Fixed cheliceral digit spinous and 10 (10) long. Palpal femora with thick plumose setae* dF*, 15 (15) long; palpal genua with filiform slightly serrate setae* dG*, 30 (25) long. Palpal tibiae with 3 smooth setae:* dTi*,* l'Ti*,* l”Ti* and long curved claw. Palpal tarsi with 4 smooth setae. Subcapitular setae* n* densely serrate, about 25 (35) long. Each branch of peritremes with barely discernible chambers, 60 (60) long. Hypostome 115 (125) long, with flattened apex.* Idiosoma* 330 (320) long and 365 (395) wide. Dorsum. Propodonotal shield reduced to small ovoid platelets present anterolaterally. On each platelet eyes and two thick, plumose, and slightly apically expanded setae about 25 long, present. Posterior to platelets 5 thick and serrate setae, 25–30 long. Anterolateral and mediolateral parts with about 40 pairs of slightly plumose setae (10–25 long) that increase in length from anterior to posterior part of idiosoma. Posterolateral parts with about 45 pairs of blunt-pointed, slightly serrate setae, 35–60 long. Venter. Anterior part with about 50 pairs of short and thick plumose setae, 10–15 long; medial part with numerous (about 140 setae) scale-like setae, about 25 long, lateral and posterior parts with longer slender and blunt-pointed setae, 45–60 long. Genital region. Genital setae represented by 3 pairs of slender blunt-pointed setae* g1*–*g4* 60 (65), 55 (55), 30 (30), and 40 (35) long, respectively. Pseudanal series represented by 11 densely serrate, flattened setae* ps*, 30–75 long.* Legs*. Coxal setation:* 1a*,* 1b*,* 2a*,* 2b*,* 3a*,* 3b*,* 3c*,* 4a*,* 4b* arranged in formula: 2–2–3–2. All coxal setae spur-like, except for simple and slightly serrate setae* 1a* and* 1b*. Leg chaetotaxy as in group 1 of Jack [6]: tibiae I–IV (5–5–5–5), genua I–IV (1–0–0–1), femora I–IV (2–2–2–2), trochanters I–IV (1–1–1–1). Setae* dTiI–IV*,* l'TiI–IV*,* l”TiI–IV*,* vGI *simple and smooth; setae* v'TiI–IV*,* v”TiI–IV*,* vGIV* simple and with barely discernible serration;* dFI–IV* simple and slightly serrate,* vFI–IV* and* vTrI–IV* spur-like with serrate apices. Setation of tarsi I: 14 setae (*ft*,* tc'*,* tc”*,* p'*,* p”*,* a'*,* a”*,* it'*,* it”*,* u'*,* u”*,* vs'*,* vs”*,* pl'*) and solenidion* ω1*; tarsi II: 10 setae (*tc'*,* tc”*,* p'*,* p”*,* a'*,* a”*,* u'*,* u”*,* vs'*,* vs”*) and* ω1*; tarsi III and IV with 10 setae each (*tc'*,* tc”*,* p'*,* p”*,* a'*,* a”*,* u'*,* u”*,* vs'*,* vs”*). Solendion* ω1* at least 2 times longer than seta* ft*. Setae* tc'*,* tc”*,* it'*,* it” *of leg I represented by euphatidia;* tc'* and* tc”* of legs II–IV,* u'* and* u”* of legs I–IV slightly serrate,* a'* and* a”* of legs I–IV filiform,* vs'* and* vs”* of legs I–IV serrate.

*Differential Diagnosis*. This new species is the most similar to* Geckobia loricata* Berlese, 1892 described from* Tarentola mauritanica* (Linnaeus) (Gekkota: Phyllodactylidae) [12]. In females of both species the eyes are present, the setation of tibiae-coxae I–IV and tarsi I–IV is the same and the scale-like setae are present in the middle of the idiosomal venter. This new species differs from* G. loricata *by the following features. In females of* G. africana*, the propodonotal shield is present in a form of small platelets on the antero-lateral margins of the idiosoma, all posterior and posterolateral setae are slender and blunt-pointed, whereas in* G. loricata*, the propodonotal shield is absent, the posterior setae are thick and serrate, and the posterolateral setae are fine-pointed and densely serrate.

***Species Group Simplex***

*Diagnosis*

*Female. Gnathosoma.* Fixed cheliceral digit present. Palpal femora with thick serrate setae* dF*, palpal genua with filiform setae* dG*.* Idiosoma* nearly circular in shape (only slightly wider than long). Propodonotal shield present.* Leg*s. Setae of tibiae I–IV (5–5–5–5), genua I–IV (1–0–0–1), femora I–IV (3–2–2–2), trochanters I–IV (1–1–1–1).

*Host Range*. Gecko species:* Hemidactylus leschenaultia* and* Underwoodisaurus milii*. 

*Species Included*.* Geckobia simplex* Hirst, 1926 and* Geckobia milli* n. sp. 

*Remarks*. The species of the* simplex* group are the most similar to the* indica* group from Gekkonidae from South Africa [5]. In females of both groups, the shape of the idiosoma is nearly circular, the arrangement of dorsal idiosomal setae is very similar, and setae* dF* are stout and* dG* filiform. The* simplex* group differs from the* indica* group by the following features. In females of the* simplex* group five setae on tibiae I–III are present and setae* l'GI*,* l”GIV*,* dFI*,* dFII* and* lFIII* are present, whereas in females of the* indica* group, four setae on tibiae I–III are present and setae* l'GI*,* l”GIV*,* dFI*,* dFII*,* lFIII* are absent. 

***Geckobia milii n. sp.***

*Type*-*Host*.* Underwoodisaurus milii* (Bory De Saint-Vincent) (Sauria: Carphodactylidae). 

*Type*-*Locality*. Australia, Far North Queensland, Cape York Peninsula, 9.VI.1914, coll. unknown. 

*Type Material*. Female holotype and 1 female paratype. Mites removed by M. Fajfer. Female holotype is deposited in the AMU (Reg. No. AMU-PTE27.1), female paratype in the ZMUC (Reg. No. R34910).

*Etymology*. The species name is derived from the species name of the host and is a noun in apposition. 

**Figure 4:**
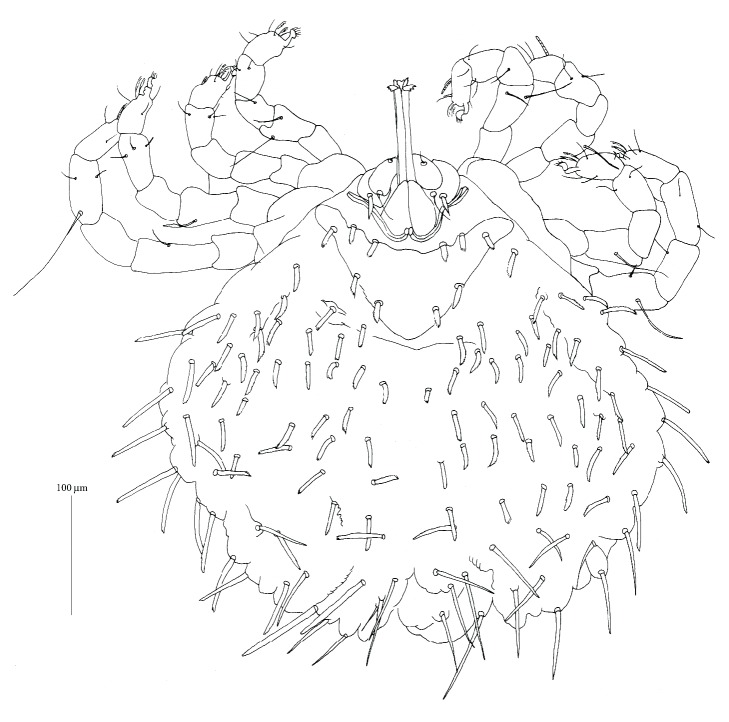
*Geckobia milii *n. sp., female in dorsal view.

**Figure 5:**
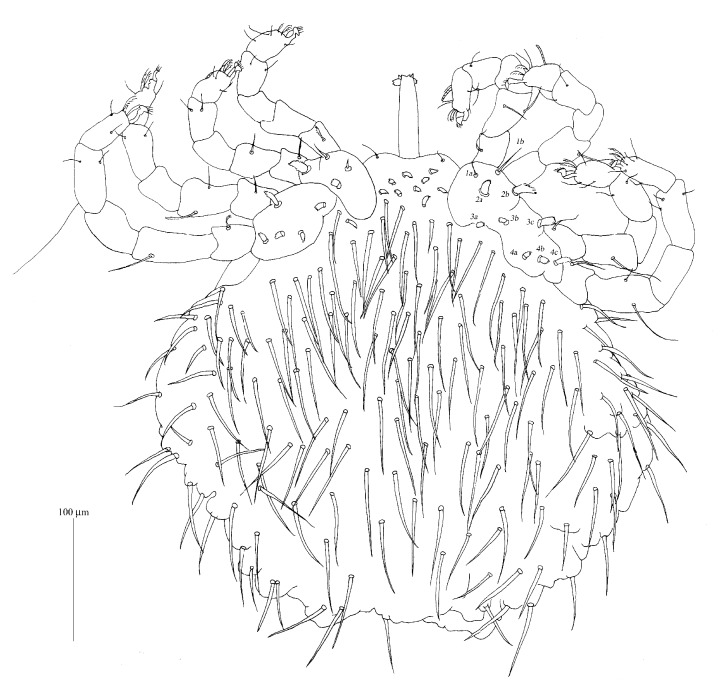
*Geckobia milii *n. sp., female in ventral view.

**Figure 6:**
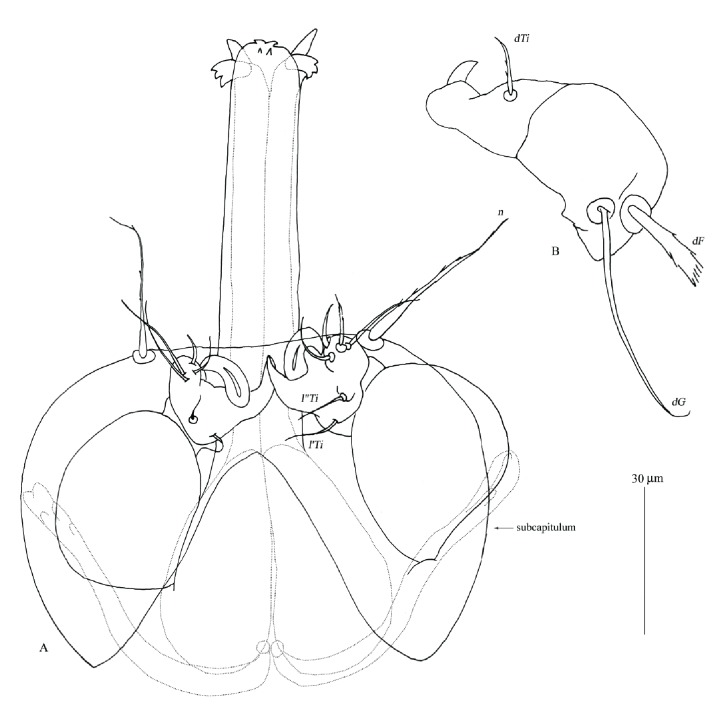
*Geckobia milii *n. sp., female, details. A, gnathosoma in ventral view; B, palps in dorsal view.

**Figure 7:**
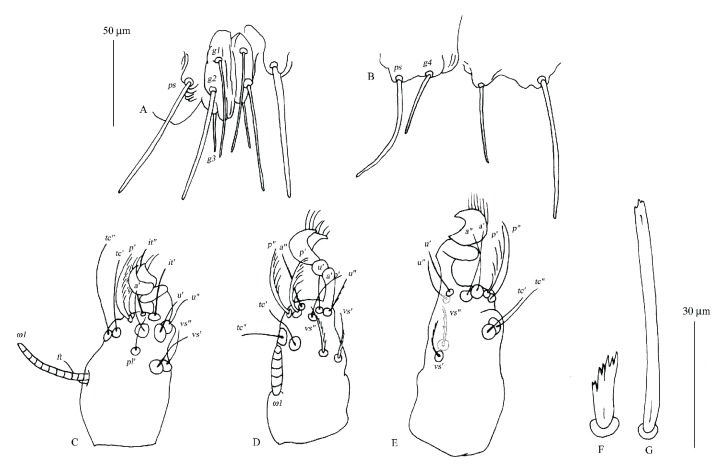
*Geckobia milii *n. sp., female, details. A, genital area in dorsal view; B, genital area in ventral view; C, tarsus I in lateral view; D, tarsus II in lateral view; E, tarsus III in lateral view; F, anterior dorsal seta; G, posterior dorsal seta.

*Description* (Figures 4–7)

*Female*.* Gnathosoma*. Chelicerae 70 (70) long; basal swollen part 45 (45) long, slender distal part 80 (80) long. Movable cheliceral digit three-pronged. Fixed cheliceral digit spinous and 5 (5) long. Palpal femora with serrate setae* dF*, 20 (25) long; palpal genua with slightly apically serrate setae* dG*, 35 (40) long. Palpal tibiae with filiform setae:* dTi*,* l'Ti*,* l”Ti* and long curved claw. Setae* l'Ti* and* l”Ti* smooth, setae* dTi* with barely discernible serration. Palpal tarsi with 4 smooth setae. Subcapitular setae* n* slightly serrate, about 25 (20) long. Each branch of peritremes with barely discernible chambers, 65 (70) long. Hypostome as in Figure 6 with small barely discernible denticles present at flattened apex, 125 (130) long.* Idiosoma* 375 (495) long and 395 (505) wide. Dorsum with triangular propodonotal shield slightly concave in anterior margin, 160 wide and 70 long. On each side of propodonotal shield 5 thick, spur-like serrate setae, 10–15 long. Eyes absent. Mediolateral and posterolateral parts of idiosoma with about 40 pairs of thick serrate setae, 20–25 long. Lateral margins with about 15 pairs of longer blunt-pointed smooth setae, 35–65 long. All venter covered with numerous (about 150 setae) smooth and fine-pointed setae, 45–65 long, except for antero-medial part of idiosoma with 8 short plumose setae, about 10 long. Genital area with 3 smooth genital setae* g1*–*g3*; setae* g1*–*g2* 60–65 long,* g3* about 25 long. Pseudanal series represented by 2 pairs of blunt-pointed smooth setae, 70–85 long.* Legs*. Coxal setation:* 1a*,* 1b*,* 2a*,* 2b*,* 3a*,* 3b*,* 3c*,* 4a*,* 4b*, arranged in formula: 2–2–3–2. All coxal setae thick, serrate and spur-like, except for simple and slightly serrate setae* 1a* and* 1b*. Leg chaetotaxy: tibiae I–IV (5–5–5–5), genua I–IV (1–0–0–1), femora I–IV (3–2–2–2), trochanters I–IV (1–1–1–1). Setae* dTiI–IV*,* l'TiI–IV*,* l”TiI–IV*,* vGI,v'TiI–IV*,* v”TiI–IV*, smooth;* dFI–IV* and* vTrI–IV* slightly serrate,* vFI–IV *and* vGIV *with barely discernible serration. Setation of tarsi I: 14 setae (*ft*,* tc'*,* tc”*,* p'*,* p”*,* a'*,* a”*,* it'*,* it”*,* u'*,* u”*,* vs'*,* vs”*,* pl'*) and solenidion* ω1*; tarsi II: 10 setae (*tc'*,* tc”*,* p'*,* p”*,* a'*,* a”*,* u'*,* u”*,* vs'*,* vs”*) and* ω1*; tarsi III and IV with 10 setae each (*tc'*,* tc”*,* p'*,* p”*,* a'*,* a”*,* u'*,* u”*,* vs'*,* vs”*). Solendion* ω1* two times longer than seta* ft*. Setae* p'* and* p”* fan-like. Setae* tc'*,* tc”*,* it'*,* it” *of leg I represented by euphatidia;* tc'* and* tc”* of legs II–IV,* u'* and* u”* of legs IV slightly serrate,* a'* and* a”* filiform,* vs'* and* vs”* serrate.

*Differential Diagnosis*. This new species is the most similar to* Geckobia simplex* Hirst, 1926 described from* Hemidactylus leschenaultia *Duméril & Bibron (Gekkonoidea: Gekkonidae) from India [13]. In females of both species, the idiosoma is nearly circular in shape and almost as wide as long, the propodonotal shield is present, the ventral setae are slender; the setation of tibia-trochanter IV and tarsi I–IV is the same. This new species differs from* G. simplex* by the following features: the propodonotal shield is triangular with slightly concave anterior margin and bears 10 setae, the eyes are absent, the dorsal anterior setae are much shorter than the posterior setae and seta* a”* of tarsi I is present. In* G. simplex*, the propodonotal shield is reniform with concave the posterior margin and bears about 36 setae, the eyes are present, the anterior dorsal setae are almost as long as the posterior setae, and seta* a”* of tarsi I is absent.

## Data Availability

The data used to support the findings of this study are available from the corresponding author upon request.
